# Targeting of AKT/ERK/CTNNB1 by DAW22 as a potential therapeutic compound for malignant peripheral nerve sheath tumor

**DOI:** 10.1002/cam4.1732

**Published:** 2018-08-15

**Authors:** Xiao‐Xiao Li, Shi‐Jie Zhang, Amy P. Chiu, Lilian H. Lo, Jian Huang, Dewi K. Rowlands, Jinhui Wang, Vincent W. Keng

**Affiliations:** ^1^ Department of Applied Biology and Chemical Technology The Hong Kong Polytechnic University Kowloon Hong Kong; ^2^ Institute of Clinical Pharmacology Guangzhou University of Chinese Medicine Guangzhou China; ^3^ Department of Medicinal Chemistry and Natural Medicine Chemistry (State‐Province Key Laboratories of Biomedicine‐Pharmaceutics of China) Harbin Medical University Harbin China; ^4^ Laboratory Animal Services Centre The Chinese University of Hong Kong Sha Tin New Territories Hong Kong

**Keywords:** AKT, apoptosis, DAW22, ERK, MPNST

## Abstract

Malignant peripheral nerve sheath tumors (MPNSTs) are an aggressive form of soft tissue neoplasm with extremely poor prognosis and no effective medical options currently available. MPNSTs can occur either sporadically or in association with the neurofibromatosis type 1 (NF1) syndrome. Importantly, activation of RAS/RAF/MEK/ERK, PI3K/AKT/mTOR, and WNT/CTNNB1 signaling pathways has been reported in both NF1‐related and late‐stage sporadic MPNSTs. In this study, we found that DAW22, a natural sesquiterpene coumarin compound isolated from *Ferula ferulaeoides (Steud.) Korov*., could inhibit cell proliferation and colony formation in five established human MPNST cancer cell lines. Further molecular mechanism exploration indicated that DAW22 could target the main components in the MPNST tumorigenic pathways: namely suppress phosphorylation of AKT and ERK, and reduce levels of non‐phospho (active) CTNNB1. Using the xenograft mouse model transplanted with human MPNST cancer cell line, daily treatment with DAW22 for 25 days was effective in reducing tumor growth. These results support DAW22 as an alternative therapeutic compound for MPNST treatment by affecting multiple signaling transduction pathways in its disease progression.

## 1 INTRODUCTION

Malignant peripheral nerve sheath tumors (MPNSTs) are the sixth most common aggressive soft tissue sarcoma derived from the Schwann cell lineage and/or its precursor, with a highly invasive property to surrounding peripheral nerves.^1^, ^2^ MPNSTs can either occur sporadically or in the context of patients with NF1 syndrome, emerging either de novo or developing from the pre‐existing benign plexiform neurofibroma.^3^ The incidence of developing sporadic MPNST is about 0.001%, while NF1‐associated MPNST is 5%‐13% for patients with NF1.^4^, ^5^ The current therapeutic options include surgical resection, adjuvant radiation, and chemotherapy.^6^ Complete surgical operation offers the best treatment outcome, but limitations exist due to the possible disruption of normal surrounding nerve‐associated tissues.^7^

In order to have a better targeted therapeutic regime, potential molecules that drive MPNST tumor development have been identified through different genetic screens as candidate targets. Cooperating genes enriched in the PI3K/AKT/mTOR, mitogen‐activated protein kinase (MAPK), and WNT/CTNNB1 transduction pathways have been implicated in MPNST disease initiation and progression, as well as the main regulators in mediating cell cycle, cell division, and cell death.^8^, ^9^, ^10^ The PI3K/AKT and MAPK pathways and their upstream receptor kinases are known to be active in MPNSTs, especially in NF1‐related MPNST patients.^11^, ^12^ RAS activation caused by *neurofibromin 1* (*NF1*) mutations induces downstream activation of the AKT/mTOR and RAF/MEK/ERK signaling pathways, whereas the canonical WNT/CTNNB1 signaling pathway has also been demonstrated to be an important genetic driver of cancer progression, and inhibition of WNT and mTOR signaling pathways could synergistically induce apoptosis in MPNST cancer cells in vitro.^13^

Therapeutic drugs used in preclinical and clinical trials for the treatment of MPNSTs currently include mTOR inhibitors and its derivatives (such as everolimus and temsirolimus), with varied response on tumor growth inhibition when combined with other candidate drugs.^14^, ^15^, ^16^ The MEK inhibitor PD0325901 was reported to reduce tumor growth and prolong survival rate, but could not induce apoptosis in tumor cells,^17^ whereas tyrosine kinase inhibitors such as imatinib, sorafenib, and pazopanib, and cell division interfering agents and HSP90 inhibitors are also under investigation. These agents, either alone or in combination with other chemicals may target multiple pathways and deter any potential cell death resistance leading to better anti‐cancer effects.^18^ Different drug combinations targeting main molecules of tumorigenic pathways are still under investigation in order to obtain improved efficacy for MPNST therapy. Meanwhile, novel small molecules inhibitors are still urgently needed to target multiple pathways and prevent cancer cell death resistance.

DAW22, a natural sesquiterpene coumarin compound isolated from the *Ferula ferulaeoides (Steud.) Korov*., has been reported to trigger glioma cell apoptosis in vitro.^19^ Here, we show that DAW22 could inhibit cell proliferation in both sporadic (STS‐26T) and NF1‐associated (S462, S462‐TY, ST8814, and T265) MPNST cell lines. This anti‐proliferative effect was caused by the induction of cell death, as cell cycle assays showed no significant difference between DAW22 treatment and vehicle control. By Western blot analyses, DAW22 was demonstrated to trigger apoptosis, reduced phosphorylation of AKT and ERK, and decreased level of active form CTNNB1. In addition, DAW22 reduced the tumor growth of STS‐26T‐transplanted cells in the xenograft mouse model. Taken together, our results identify DAW22 as a promising alternative therapeutic compound for the treatment of MPNST.

## 2 MATERIALS AND METHODS

### 2.1 Purification of DAW22 from the *Ferula ferulaeoides (Steud.) Korov*

DAW22 was isolated from the root of the *Ferula ferulaeoides (Steud.) Korov*. according to previous methods.^20^ The structure was determined using nuclear magnetic resonance spectroscopy and the purity of the compound was higher than 95%, which was identified by high‐performance liquid chromatography.

### 2.2 AKT inhibitor AZD5363

AKT inhibitor AZD5363 was prepared as a 100 mmol/L stock solution in DMSO.

### 2.3 Cell culture

MPNST cell lines including STS‐26T,^21^ ST8814,^22^ S462,^23^ T265,^24^ and S462‐TY^25^ were cultured in Minimum Essential Media (MEM, Thermo Fisher Scientific, Massachusetts, USA) supplemented with 10% fetal bovine serum (Thermo Fisher Scientifi) and Antibiotic‐Antimycotic (1×) (Thermo Fisher Scientific) and maintained under standard conditions of 37°C in a humidified atmosphere with 5% CO_2_. The identity of these cell lines was verified by short tandem repeats profile comparison. All the cell lines were also tested and found to be negative for mycoplasma contamination using MycoFluor^™^ Mycoplasma Detection Kit (Thermo Fisher Scientific).

### 2.4 Cell proliferation

MPNST cell lines were plated onto 96‐well plates at a concentration of 6000 cells per well. After 24 hours, cells were treated with either vehicle control or designated doses of DAW22 for 48 hours. The cell viability was quantified by CellTiter 96 AQueous One Solution Cell Proliferation Assay (MTS) (Promega, Wisconsin, USA). The cell viability assays were performed and analyzed using the Opera Phenix high‐content imaging system (PerkinElmer, Massachusetts, USA).

### 2.5 Colony formation assay

MPNST cell lines were seeded onto six‐well plates at a density of 1000 cells per well. After 24 hours, the medium was removed and replaced with either vehicle control or DAW22‐containing medium for 2 weeks to allow for colony growth. Following 2 weeks, media were removed and cells were fixed with 4% paraformaldehyde in phosphate‐buffered saline (PBS) for 15 minutes and stained with 0.1% crystal violet solution in PBS for 10 minutes, followed by repeated washing with PBS.

### 2.6 Cell cycle assay

MPNST cell lines were seeded onto six‐well plates, followed by treatment with an indicated concentration of DAW22 for 24 hours. Next, cells were fixed with 70% ice‐cold ethanol in PBS, stained with propidium iodide (PI, 50 μg/mL, Thermo Fisher Scientific), and analyzed by flow cytometer.

### 2.7 Western blot analyses

Protein was isolated from DAW22‐treated MPNST cell lines using the Qproteome Mammalian Protein Prep Kit (QIAGEN, Hilden, Germany) following the manufacturer's instructions. Concentrations of protein were determined by Bradford Protein Assay (Bio‐Rad, California, USA) followed by denaturation as described by the manufacturer. Protein was separated on a 12% SDS‐PAGE gel and transferred to polyvinylidene difluoride (PVDF) membrane (Millipore, Massachusetts, USA). Protein on the membrane was first incubated with indicated primary antibodies at 4°C overnight, followed by corresponding secondary antibodies’ incubation at room temperature for 1 hour. Targeted proteins were detected using a horseradish peroxidase‐conjugated chemiluminescent kit (Millipore). AKT (#2920), phospho‐AKT (#4060), ERK (#4695), phospho‐ERK (#4370), poly(ADP‐ribose) polymerase 1 (PARP) (#9532), Non‐phospho (active) CTNNB1 (#8814), caspase 3 (CASP3), and ACTB (#4970), with the exception for CASP3 that was purchased from Santa Cruz Biotechnology (Texas, USA), remaining primary antibodies were from Cell Signaling Technology (Massachusetts, USA). ACTB was used as the loading control.

### 2.8 Xenograft mouse model

Six‐week‐old immunocompromised nude mice (The Laboratory Animal Services Centre, The Chinese University of Hong Kong, Hong Kong) were anesthetized with ketamine and each flank injected subcutaneously with 2 × 10^6^ STS‐26T cells in 0.1 mL PBS containing 50% Matrigel (Corning, New York, USA). One week later, mice were randomly divided into two groups and treated with either vehicle or DAW22 at a dose of 60 mg/kg/d for 25 days by daily intraperitoneal injection. Body weights and tumor sizes were measured every 3 days. At the experimental end point (25 days) and upon necropsy, tumor sizes and weights were measured for each mouse. All animal studies were approved by the appropriate ethics committee and performed in accordance with the ethical standards stipulated by both The Hong Kong Polytechnic University and The Chinese University of Hong Kong.

### 2.9 Hematoxylin and eosin staining

Tissues were carefully removed from the sacrificed animal, weighed, washed, and placed in cold PBS. Formalin‐fixed paraffin‐embedded sections from various tissues were sectioned at 5 μm using a standard microtome (Leica Biosystems, Wetzlar, Germany), mounted, and heat‐fixed onto glass slides. Tissue section slides were either processed and stained with hematoxylin and eosin (HE) using standard protocols.

### 2.10 Statistical analyses

Raw data were analyzed using GraphPad Software Prism (Version 6, California, USA), and resulting values were expressed as mean ± standard error of the mean (SEM). Student's *t* test and ANOVA in Prism were used for statistical analyses. Value of *P *<* *0.05 was considered as statistically significant.

## 3 RESULTS

### 3.1 DAW22 inhibits cell proliferation in both sporadic and NF1‐related MPNST cell lines

**Figure 1:**
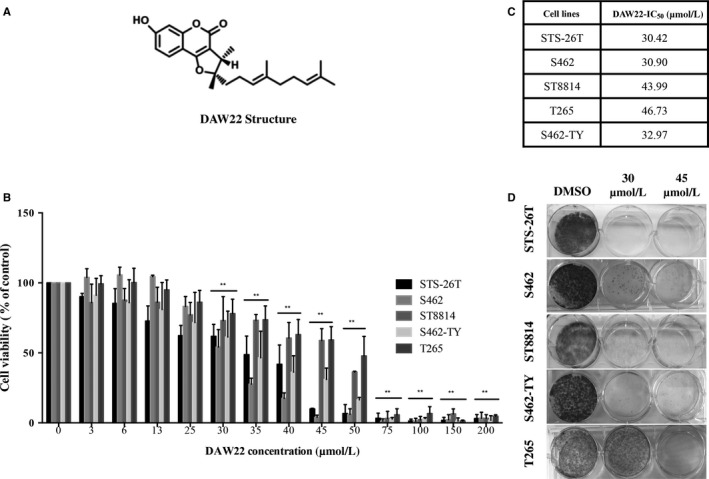
DAW22 inhibited cell proliferation of sporadic and NF1‐related MPNST cell lines. A, Chemical structure of DAW22. B, MTS cell viability assay was performed on MPNST cell lines after exposure to indicated concentrations of DAW22 for 48 h. C, Concentrations of DAW22 inducing 50% growth inhibition (IC_50_) in sporadic and NF1‐related MPNST cell lines. ST8814 and T265 cell lines were more resistant to DAW22, compared with S462, S462‐TY, and STS‐26T cell lines. DAW22 IC_50_ values were calculated using GraphPad Prism (Version 6). D, Colony formation analyses of five MPNST cell lines after DAW22 treatment. These five cell lines were seeded on six‐well plate at a density of 1000 cells per well, treated with 30 and 45 μmol/L DAW22 for 2 wk, and stained with crystal violet. Values were presented as mean ± SEM of three independent experiments. **P <* *0.01, compared with vehicle control.

The therapeutic potential of DAW22, structure shown in Figure 1A, was evaluated using different human MPNST cancer cell lines. The genetic information of the different human MPNST cancer cell lines used in this study is summarized in Table 1.^26^, ^27^, ^28^ Different concentrations of DAW22 were exposed to a panel of five MPNST cell lines for 48 hours: sporadic MPNST cell line STS‐26T and four NF1‐associated MPNST cell lines S462, S462‐TY, ST8814, and T265.

**Table 1:** Genetic background information of human MPNST cancer cell lines. Genetic background information about the MPNST cell lines used in this study. Expression levels of major genetic components in PI3K/AKT pathway were compared with an immortalized human Schwann cell line, HSC1λ. Data were compiled via literature review.^26^, ^27^, ^28^ Major genetic components involved in WNT/CTNNB1 pathway were performed by our laboratory

Cell lines	*NF1*	*TP53*	*RAS‐GTP*	PI3K/AKT		WNT/CTNNB1				
				*PTEN*	*AKT‐mTOR*	*GSK3B*	*CDKN2A*	*MARK2*	*PPP2R2A*	*CREBBP*
STS‐26T	+/+	Absent	Lower	Normal	Higher	Lower	Lower	Higher	Lower	Lower
T265	−/−	Normal	Higher	Lower	Higher	Lower	Absent	Normal	Lower	Lower
ST8814	−/−	Normal	Higher	Lower	Higher	Lower	Absent	Higher	Lower	Higher
S462	−/−	Mutant overexpression	Higher	Normal	Lower	Lower	Absent	Higher	Higher	Higher
S462‐TY	−/−	Mutant overexpression	NA	NA	Lower	Higher	Absent	Higher	Higher	Higher

Cell proliferation rates were determined, and the concentrations that caused a 50% inhibition of cell viability (IC_50_) in these five cell lines ranged from 30.42 to 46.73 μmol/L (Figure 1B,C). To further study the anti‐proliferative effect of DAW22, colony formation assay was conducted to observe whether DAW22 could affect cellular attachment, survival, and proliferation. DAW22 treatment suppressed the formation of MPNST cancer cell colonies (Figure 1D).

### 3.2 DAW22 inhibits cell proliferation in MPNST cell lines through the induction of apoptosis

**Figure 2:**
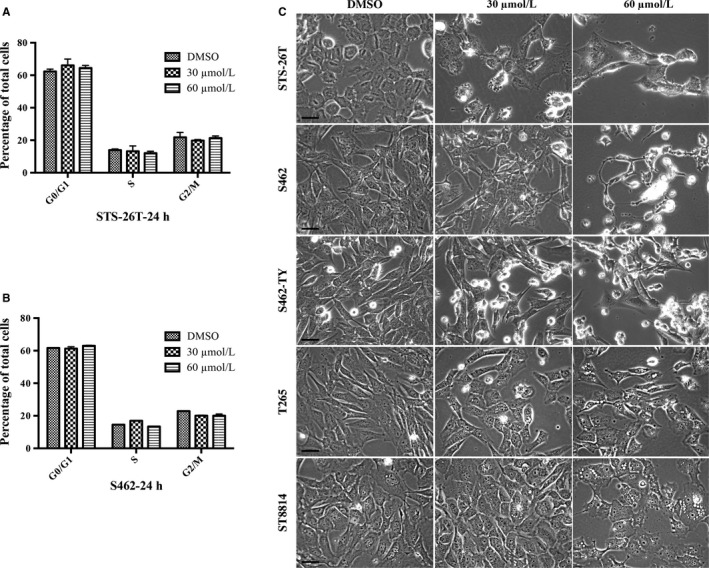
Cell cycle analyses and morphological changes in DAW22‐treated MPNST cell lines. Negative effect of DAW22 on cell cycle of MPNST cancer cell lines STS‐26T (A) and S462 (B). Cells were seeded on six‐well plate and treated with 30 and 60 μmol/L of DAW22 for 24 h. Cell cycle was analyzed by propidium iodide staining and flow cytometry. C, Morphological changes observed in DAW22‐treated MPNST cell lines. Cell shrinkage, rounding, and loss of adhesion in culture medium were observed, indicating cellular damage or cell death. Scale bars, 10 μm.

**Figure 3:**
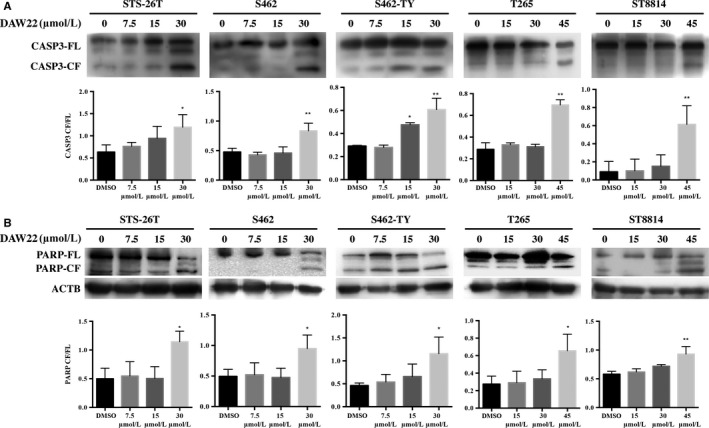
Induced apoptosis in DAW22‐treated MPNST cells lines. Cells were exposed with different concentrations of DAW22 for 48 h. Western blot analyses were performed to detect levels of both full‐length (FL) and cleaved (CF) versions of CASP3 (A) and PARP (B). Quantitative analyses of CF relative to its FL shown in (A) and (B). Values were expressed as mean ± SEM of three independent blots. *P <* *0.05; **P <* *0.01, compared with vehicle control. ACTB loading only shown in (B). Western blot images shown in (A) and (B) were representative results showing similar trend from at least three independent experiments.

Inhibition of cell proliferation is either caused by cell cycle arrest or the induction of programmed cell death. Cell cycle assay was performed by flow cytometry using cells that were treated with DAW22 at 30 and 60 μmol/L concentrations for 24 hours. DAW22 could not induce cell cycle arrest, with no significant differences in G2/M phase for two representative cell lines (Figures 2A,B and S1). However, MPNST cell lines exposed with either concentrations of DAW22 showed obvious morphological phenotype such as cell shrinkage, rounding, and loss of adhesion in culture medium, indicating cellular damage and death (Figure 2C). Apoptotic budding was observed in STS‐26T cells exposed with 30 μmol/L DAW22 for 48 hours (Figure S2). To confirm the cell death induced by DAW22 in these MPNST cell lines was due to apoptosis, total levels of CASP3 and PARP as well as their cleaved forms were analyzed by Western blot analyses. Exposure of DAW22 for 48 hours induced significant increase in cleaved CASP3 and PARP in a dose‐dependent manner (Figure 3A,B). The induction of cleaved CASP3 and PARP in each cell line occurred around their IC_50_ concentration, which was consistent with the cell proliferative inhibition data (Figure 1B,C). Furthermore, these apoptotic effects were induced by DAW22 in a time‐dependent manner (Figure S3). DAW22 concentration used for each cell line corresponded with their IC_50_: 30 μmol/L in STS‐26T, S462, and S462‐TY cells; 45 μmol/L in ST8814 and T265 cells. Taken together, these data support that DAW22 could induce programmed cell death in MPNST cell lines by eliciting apoptosis.

### 3.3 DAW22 reduced phosphorylation of AKT, ERK, and nonphospho (active) CTNNB1 in MPNST cell lines

**Figure 4:**
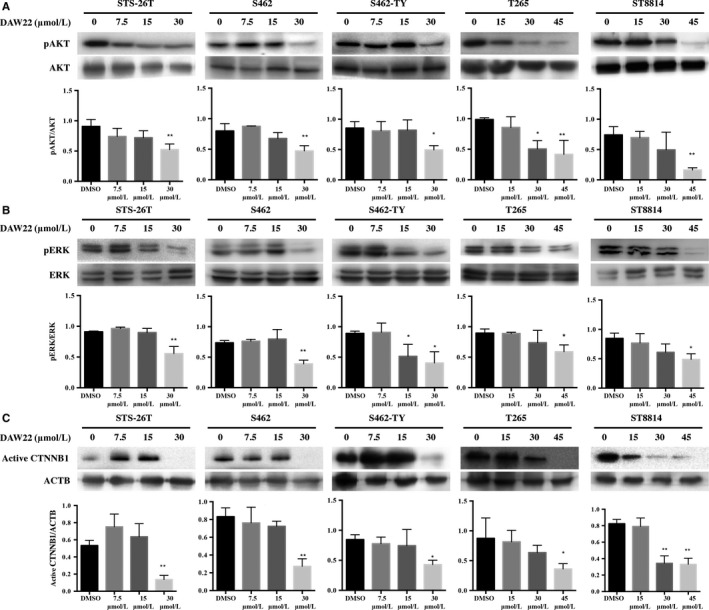
DAW22 reduced phosphorylation of AKT, ERK, and non‐phospho (active) CTNNB1 in MPNST cell lines. Cells were treated with different concentrations of DAW22 for 48 h. Levels of phosphorylated AKT/ERK, total AKT/ERK, and active CTNNB1 were detected by Western blot analyses, as shown in (A), (B), and (C). Quantitative analyses of phosphorylated protein relative to its total protein shown in (A) and (B), while active CTNNB1 relative to ACTB was shown in (C). Values were expressed as mean ± SEM of three independent blots. *P <* *0.05; **P <* *0.01, compared with vehicle control. ACTB loading only shown in (C). Western blot images shown in (A), (B), and (C) were representative results showing similar trend from at least three independent experiments.

It has been widely reported that PI3K/AKT/mTOR, MAPK, and WNT/CTNNB1 pathways all play major roles in MPNST tumor initiation and progression. To test the potential effects of DAW22 on these major pathway regulators, Western blot analyses were used to determine changes in each signaling pathway. AKT was found to be overexpressed as well as overactivated by phosphorylation in MPNST cell lines. However, DAW22 could remarkably induce a reduction in phosphorylated AKT in both sporadic and NF1‐related MPNST cell lines (Figure 4A). To evaluate whether DAW22 could also affect the MAPK pathway, total and phosphorylated ERK were analyzed. Phosphorylation of ERK was dramatically reduced when treated with DAW22 at their IC_50_ concentrations (Figure 4B). Reduced phosphorylation of AKT and ERK was also observed at different time points when administrated with DAW22 at 30 μmol/L DAW22 in STS‐26T, S462, and S462‐TY cells, while 45 μmol/L DAW22 in ST8814 and T265 cells (Figure S4). DAW22 also reduced the levels of non‐phosphorylated active form of CTNNB1 (Figure 4C). In addition, AKT inhibitor AZD5363 induced apoptosis in MPNST cell lines but at a much higher dose compared with DAW22 (Figure S5).

### 3.4 DAW22 treatment delayed the growth of STS‐26T cells in xenograft transplant experiments

**Figure 5:**
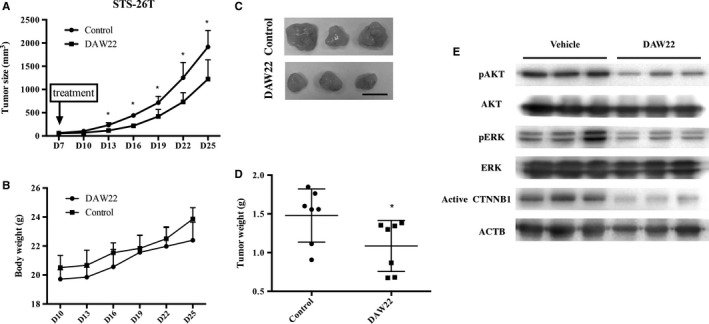
In vivo anti‐cancer effect of DAW22 on STS‐26T‐transplanted xenograft mouse model. A, Quantitative analyses of tumor volume in mice from vehicle‐treated and DAW22‐treated groups. Six‐week‐old nude mice were engrafted with STS‐26T cells and treated with DAW22 (60 mg/kg/d) 1 wk after transplantation. DAW22 was introduced by intraperitoneal injection once daily for 25 d. B, Body weights from both vehicle‐treated and DAW22‐treated groups showed no significant differences for the entire treatment period of 25 d. C, Representative images of STS‐26T subcutaneous tumor xenografts at experimental end point. Scale bar, 1 cm. D, Significant reduction in tumor weights from DAW22‐treated group compared with vehicle‐treated animals. Values were expressed as mean ± SEM; *P <* *0.05. E, Protein was isolated from transplanted xenograft tumors from both vehicle‐treated and DAW22‐treated groups. Expression levels of phosphorylated AKT/ERK, total AKT/ERK, and active CTNNB1 were evaluated by Western blot analyses.

**Figure 6:**
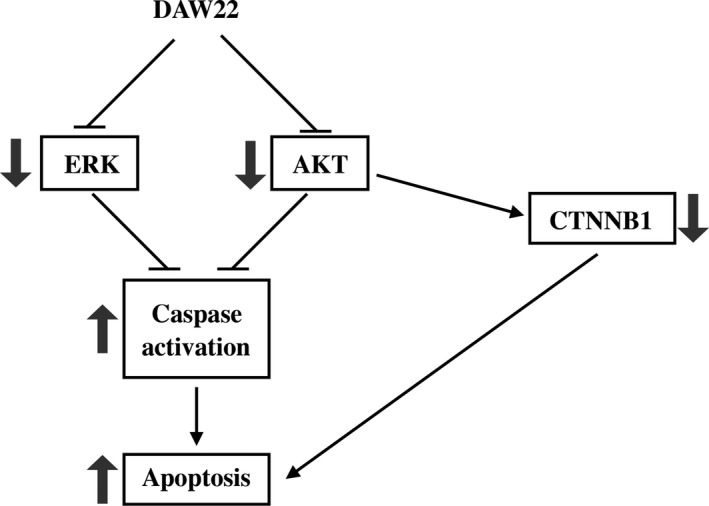
DAW22 targets multiple signaling pathways involved in MPNST disease progression. DAW22 inhibits expression of phosphorylated ERK, AKT, and non‐phosphorylated (active) CTNNB1. This contributes to the induction of apoptosis by DAW22 in MPNST in vitro and in vivo.

To determine whether DAW22 could impede xenograft tumor growth, DAW22 was administered to immunocompromised nude mice transplanted subcutaneously with STS‐26T cells. One week after cell transplantation, mice were injected intraperitoneally with DAW22 at a daily dose of 60 mg/kg/d for 4 weeks and tumor volume recorded every 3 days. DAW22 administration significantly inhibited the tumor xenografts compared with vehicle control group (Figure 5A,C). During the treatment period, there was no obvious loss of body weight in DAW22‐treated group, indicating no gross toxicity effect caused by DAW22 (Figure 5B). HE staining performed on tissues (liver, kidney, heart, lung, and spleen) taken from both vehicle‐treated and DAW22‐treated animals showed no adverse effect (Figure S6). At the experimental end point (25 days posttreatment), the mean tumor weights in DAW22 treatment group were 1.086 ± 0.1247 g, compared with 1.478 ± 0.1296 g for control (*P *=* *0.0499) (Figure 5D). In order to confirm that AKT, ERK, and CTNNB1 were targets of DAW22, the protein expression level of phosphorylated AKT, ERK, and active CTNNB1 in xenografted tumors was analyzed (Figure 6). Reduction in phosphorylated AKT, ERK, and active CTNNB1 was observed in DAW22‐treated tumors compared with vehicle‐treated group (Figure 5E).

## 4 DISCUSSION

With a high rate of metastases and extremely poor prognosis, MPNSTs represents one of the most difficult‐to‐cure sarcoma. Currently, there are no effective drugs for the treatment of MPNST and surgical resection remains the most effective means of therapy, but this method is limited due to the close proximity of the affected peripheral nerves with other tissues. Better therapeutic regimes for MPNST require a greater understanding of the genetic mechanisms associated with the disease. As precision medicine becomes more important for cancer therapy, identification of accurate therapeutic targets and discovery of specific drugs to control cancer development are becoming ever more critical.

In our study, DAW22, a compound isolated from the plant *Ferula ferulaeoides (Steud.) Korov*., inhibited cell proliferation in both sporadic and NF1‐related MPNST cell lines at varied doses. ST8814 and T265 cell lines have a IC_50_ of about 45 μmol/L, compared with STS‐26T, S462, and S462‐TY cell lines, where the IC_50_s were around 30 μmol/L. The differences might be caused by their distinct genetic backgrounds (Table 1). The higher IC_50_ of ST8814 and T265 may result from their normal expression of *tumor protein p53* (*TP53*) tumor suppressor gene,^29^ higher RAS‐GTP level caused by *NF1* deficiency, and activated AKT‐mTOR signaling,^30^ compared with S462 cells. S462‐TY cell line has a similar IC_50_ concentration as S462 cells, as S462‐TY was derived from a xenograft passage of S462.^25^ The *TP53* expression in STS‐26T cells was completely absent,^31^ which may have contributed to its relative low IC_50_ concentration. Cell cycle was not influenced by DAW22 based on our cell cycle assay results (Figures 2A and S1). The apoptotic budding in STS‐26T cells was observed, which suggested that DAW22 could induce apoptosis in MPNST cell lines. Consistent with the apoptotic budding observation, the cleaved CASP3 and PARP increased under DAW22 treatment in MPNST cell lines, which confirm that DAW22 could indeed trigger apoptotic cell death. The concentration of DAW22 that elicited apoptosis in each cell line was close to their IC_50_s. Interestingly, DAW22 could induce apoptosis 12 hours after treatment in STS‐26T, S462‐TY, and S462 cell lines at 30 μmol/L, while it was after 24 hours in ST8814 and T265 cell lines at 45 μmol/L, which further suggests that varying genetic backgrounds could contribute to distinct cellular responses. Interestingly, cytoplasmic vacuolization was also observed in DAW22‐treated MPNST cancer cell lines (data not shown). This could be paraptosis‐like cell death, which could further contribute to the anti‐cancer effect. However, the molecular mechanism(s) associated with paraptosis remains to be elucidated.

Accumulating evidence indicated several pathways are highly related with MPNST transformation ability. NF1‐related MPNST cancer patients have activated RAS signaling, which subsequently cause activation of PI3K/AKT/mTOR and MAPK pathways. Sporadic MPNST patients also showed mutations in these pathways at the advanced disease stages. Moreover, significant activation of WNT/CTNNB1 pathway has been shown to drive human Schwann cell transformation and tumor maintenance in development of MPNST. The important roles of these pathways were further validated using inhibitors targeting AKT, mTOR, MEK, and WNT pathways either singly or in combinations.^13^, ^16^, ^30^, ^32^, ^33^, ^34^

Here, we demonstrated that DAW22 inhibited phosphorylation of AKT, ERK, and active form of CTNNB1. The data indicated that DAW22 could target multiple signaling pathways involved in MPNST disease progression (Figure 6). In addition, AKT has been reported to regulate CTNNB1 phosphorylation and degradation in tumor invasion and development. The effect of AKT on CTNNB1 phosphorylation could be either direct phosphorylation^35^ or indirectly regulation via the GSK3β, resulting in the accumulation of CTNNB1.^36^ This interaction between CTNNB1 and AKT conferred resistance to AKT inhibitor in colon cancer.^37^ This could explain the higher IC_50_s of AKT inhibitor AZD5363 in MPNST cancer cell lines (Figure S5). As AKT, ERK, and CTNNB1 are currently the most important components in the transduction pathways for MPNST disease progression, DAW22 can be used as a potential therapeutic alternative in fighting against cancer, especially in AKT‐resistant cancer types. STS‐26T, S462, and S462‐TY were previously used as transplanted cell strains for MPNST xenograft experiments.^38^, ^39^, ^40^ In advanced MPNST stage, NF1‐associated patients cannot be distinguished from sporadic MPNST patients, indicating that they both ultimately share a similar genetic profile.^41^ Therefore, in our study, the sporadic MPNST STS‐26T cells were used to establish the xenograft MPNST cancer model. We found that DAW22 alone delayed tumor development in STS‐26T transplanted xenograft mouse model, resulting in lower tumor growth rate and decreased tumor weight.

In summary, our current study showed that DAW22 inhibited both sporadic and NF1‐related MPNST cancer cell proliferation and induced apoptosis by targeting AKT, ERK, and CTNNB1 pathways. In addition, DAW22 delayed tumor growth of STS‐26T cell transplanted in xenograft mice, providing strong evidence for DAW22 as a potential novel alternative therapeutic treatment for patients with MPNST.

## CONFLICT OF INTEREST

The authors have no conflict of interest.

## References

[1] James AW , Shurell E , Singh A , Dry SM , Eilber FC . Malignant peripheral nerve sheath tumor. Surg Oncol Clin N Am. 2016;25(4):789‐802. 27591499 10.1016/j.soc.2016.05.009

[2] Consortium ECTS . Identification and characterization of the tuberous sclerosis gene on chromosome 16. Cell. 1993;75(7):1305‐1315. 8269512 10.1016/0092-8674(93)90618-z

[3] Ferner RE , Gutmann DH . International Consensus Statement on Malignant Peripheral Nerve Sheath Tumors in Neurofibromatosis 1. *Canc Res* 2002;62:1573–1577. 11894862

[4] Evans DGR , Baser ME , McGaughran J , Sharif S , Howard E , Moran A . Malignant peripheral nerve sheath tumours in neurofibromatosis 1. J Med Genet. 2002;39(5):311‐314. 12011145 10.1136/jmg.39.5.311 PMC1735122

[5] Farid M , Demicco EG , Garcia R , et al. Malignant peripheral nerve sheath tumors. Oncologist. 2014;19(2):193‐201. 24470531 10.1634/theoncologist.2013-0328 PMC3926794

[6] Widemann BC . Current status of sporadic and neurofibromatosis type 1‐associated malignant peripheral nerve sheath tumors. Curr Oncol Rep. 2009;11(4):322‐328. 19508838 10.1007/s11912-009-0045-z PMC6689400

[7] Packer RJ , Rosser T . Review ArticLe: Therapy for Plexiform Neurofibromas in Children With Neurofibromatosis 1: An Overview. J Child Neurol. 2002;17(8):638‐641. 12403563 10.1177/088307380201700816

[8] Rahrmann EP , Watson AL , Keng VW , et al. Forward genetic screen for malignant peripheral nerve sheath tumor formation identifies new genes and pathways driving tumorigenesis. Nat Genet. 2013;45(7):756‐766. 23685747 10.1038/ng.2641 PMC3695033

[9] Luscan A , Masliah‐Planchon J , Laurendeau I , et al. The activation of the WNT signalling pathway is a hallmark in Neurofibromatosis type 1 tumorigenesis. Clin Cancer Res. 2013;20:358‐371. 24218515 10.1158/1078-0432.CCR-13-0780

[10] Endo M , Yamamoto H , Setsu N , et al. Prognostic significance of AKT/mTOR and MAPK pathways and anti‐tumor effect of mTOR inhibitor in NF1‐related and sporadic malignant peripheral nerve sheath tumors. Clin Cancer Res. 2012;19:450‐461. 23209032 10.1158/1078-0432.CCR-12-1067

[11] Holtkamp N , Okuducu AF , Mucha J , et al. Mutation and expression of PDGFRA and KIT in malignant peripheral nerve sheath tumors, and its implications for imatinib sensitivity. Carcinogenesis. 2005;27(3):664‐671. 16357008 10.1093/carcin/bgi273

[12] Patwardhan PP , Surriga O , Beckman MJ , et al. Sustained inhibition of receptor tyrosine kinases and macrophage depletion by PLX3397 and rapamycin as a potential new approach for the treatment of MPNSTs. Clin Cancer Res. 2014;20(12):3146‐3158. 24718867 10.1158/1078-0432.CCR-13-2576 PMC4060793

[13] Watson AL , Rahrmann EP , Moriarity BS , et al. Canonical Wnt/β‐catenin signaling drives human Schwann cell transformation, progression, and tumor maintenance. Cancer Discov. 2013;3(6):674‐689. 23535903 10.1158/2159-8290.CD-13-0081 PMC3679355

[14] Widemann BC , Meyer CF , Cote GM , et al. SARC016: Phase II Study of Everolimus in comBination With Bevacizumab in Sporadic and Neurofibromatosis Type 1 (NF1) Related Refractory Malignant Peripheral Nerve Sheath Tumors (MPNST). *J Clin Oncol* 2016;34:suppl; abstr 11053. 10.1155/2019/7656747 PMC6681622 31427883

[15] Yamashita A , Baia G , Ho J , et al. Preclinical evaluation of the combination of mTOR and proteasome inhibitors with radiotherapy in malignant peripheral nerve sheath tumors. J Neurooncol. 2014;118(1):83‐92. 24668609 10.1007/s11060-014-1422-5 PMC4059766

[16] Ghadimi MP , Lopez G , Torres KE , et al. Targeting the PI3K/mTOR axis, alone and in combination with autophagy blockade, for the treatment of malignant peripheral nerve sheath tumors. Mol Cancer Ther. 2012;11(8):1758‐1769. 22848094 10.1158/1535-7163.MCT-12-0015 PMC3416967

[17] Jessen WJ , Miller SJ , Jousma E , et al. MEK inhibition exhibits efficacy in human and mouse neurofibromatosis tumors. J Clin Investig. 2013;123(1):340‐347. 23221341 10.1172/JCI60578 PMC3533264

[18] Kim A , Pratilas CA . The promise of signal transduction in genetically driven sarcomas of the nerve. Exp Neurol. 2017;299:317‐325. 28859862 10.1016/j.expneurol.2017.08.014

[19] Zhang L , Tong X , Zhang J , Huang J , Wang J . DAW22, a natural sesquiterpene coumarin isolated from *Ferula ferulaeoides* (*Steud*.) *Korov*. that induces C6 glioma cell apoptosis and endoplasmic reticulum (ER) stress. Fitoterapia. 2015;103:46‐54. 25776007 10.1016/j.fitote.2015.03.010

[20] Meng H , Li G , Huang J , Zhang K , Wang H , Wang J . Sesquiterpene coumarin and sesquiterpene chromone derivatives from *Ferula ferulaeoides (Steud.) Korov* . Fitoterapia. 2013;86:70‐77. 23422224 10.1016/j.fitote.2013.02.002

[21] Dahlberg W , Little J , Fletcher J , Suit H , Okunieff P . Radiosensitivity in vitro of human soft tissue sarcoma cell lines and skin fibroblasts derived from the same patients. Int J Radiat Biol. 1993;63(2):191‐198. 8094415 10.1080/09553009314550251

[22] Fletcher JA , Kozakewich HP , Hoffer FA , et al. Diagnostic relevance of clonal cytogenetic aberrations in malignant soft‐tissue tumors. N Engl J Med. 1991;324(7):436‐443. 1988828 10.1056/NEJM199102143240702

[23] Frahm S , Mautner V‐F , Brems H , et al. Genetic and phenotypic characterization of tumor cells derived from malignant peripheral nerve sheath tumors of neurofibromatosis type 1 patients. Neurobiol Dis. 2004;16(1):85‐91. 15207265 10.1016/j.nbd.2004.01.006

[24] Badache A , De Vries GH . Neurofibrosarcoma‐derived Schwann cells overexpress platelet‐derived growth factor (PDGF) receptors and are induced to proliferate by PDGF BB. J Cell Physiol. 1998;177(2):334‐342. 9766530 10.1002/(SICI)1097-4652(199811)177:2<334::AID-JCP15>3.0.CO;2-9

[25] Mahller YY , Vaikunth SS , Ripberger MC , et al. Tissue inhibitor of metalloproteinase‐3 via oncolytic herpesvirus inhibits tumor growth and vascular progenitors. Can Res. 2008;68(4):1170‐1179. 10.1158/0008-5472.CAN-07-2734 PMC2855837 18281493

[26] de Toledo SM , Azzam EI , Dahlberg WK , Gooding TB , Little JB . ATM complexes with HDM2 and promotes its rapid phosphorylation in a p53‐independent manner in normal and tumor human cells exposed to ionizing radiation. Oncogene. 2000;19(54):6185‐6193. 11175332 10.1038/sj.onc.1204020

[27] Kraniak JM , Sun D , Mattingly RR , Reiners JJ , Tainsky MA . The role of neurofibromin in N‐Ras mediated AP‐1 regulation in malignant peripheral nerve sheath tumors. Mol Cell Biochem. 2010;344(1–2):267‐276. 20680410 10.1007/s11010-010-0551-1 PMC3809002

[28] Mahller YY , Rangwala F , Ratner N , Cripe TP . Malignant peripheral nerve sheath tumors with high and low Ras‐GTP are permissive for oncolytic herpes simplex virus mutants. Pediatr Blood Cancer. 2006;46(7):745‐754. 16124003 10.1002/pbc.20565

[29] Li Y , Rao PK , Wen R , et al. Notch and Schwann cell transformation. Oncogene. 2004;23(5):1146‐1152. 14762442 10.1038/sj.onc.1207068

[30] Watson AL , Anderson LK , Greeley AD , et al. Co‐targeting the MAPK and PI3K/AKT/mTOR pathways in two genetically engineered mouse models of schwann cell tumors reduces tumor grade and multiplicity. Oncotarget. 2014;5(6):1502. 24681606 10.18632/oncotarget.1609 PMC4039227

[31] Miller SJ , Rangwala F , Williams J , et al. Large‐scale molecular comparison of human schwann cells to malignant peripheral nerve sheath tumor cell lines and tissues. Can Res. 2006;66(5):2584‐2591. 10.1158/0008-5472.CAN-05-3330 16510576

[32] Zou CY , Smith KD , Zhu Q‐S , et al. Dual targeting of AKT and mammalian target of rapamycin: a potential therapeutic approach for malignant peripheral nerve sheath tumor. Mol Cancer Ther. 2009;8(5):1157‐1168. 19417153 10.1158/1535-7163.MCT-08-1008

[33] Ahsan S , Ge Y , Tainsky MA . Combinatorial therapeutic targeting of BMP2 and MEK‐ERK pathways in NF1‐associated malignant peripheral nerve sheath tumors. Oncotarget. 2016;7(35):57171. 27494873 10.18632/oncotarget.11036 PMC5302981

[34] Ambrosini G , Cheema HS , Seelman S , et al. Sorafenib inhibits growth and mitogen‐activated protein kinase signaling in malignant peripheral nerve sheath cells. Mol Cancer Ther. 2008;7(4):890‐896. 18413802 10.1158/1535-7163.MCT-07-0518 PMC3267321

[35] Fang D , Hawke D , Zheng Y , et al. Phosphorylation of β‐catenin by AKT promotes β‐catenin transcriptional activity. J Biol Chem. 2007;282(15):11221‐11229. 17287208 10.1074/jbc.M611871200 PMC1850976

[36] Nusse R . Wnt signaling in disease and in development. Cell Res. 2005;15(1):28‐32. 15686623 10.1038/sj.cr.7290260

[37] Tenbaum SP , Ordóñez‐Morán P , Puig I , et al. β‐catenin confers resistance to PI3K and AKT inhibitors and subverts FOXO3a to promote metastasis in colon cancer. Nat Med. 2012;18(6):892. 22610277 10.1038/nm.2772

[38] Wu J , Patmore DM , Jousma E , et al. EGFR–STAT3 signaling promotes formation of malignant peripheral nerve sheath tumors. Oncogene. 2014;33(2):173. 23318430 10.1038/onc.2012.579 PMC3923530

[39] Ghadimi MP , Lopez G , Torres KE , et al. Targeting the PI3K/mTOR axis, alone and in combination with autophagy blockade, for the treatment of malignant peripheral nerve sheath tumors. Mol Cancer Ther. 2012;11:1758‐1769. 22848094 10.1158/1535-7163.MCT-12-0015 PMC3416967

[40] Demestre M , Terzi MY , Mautner V , Vajkoczy P , Kurtz A , Piña AL . Effects of pigment epithelium derived factor (PEDF) on malignant peripheral nerve sheath tumours (MPNSTs). J Neurooncol. 2013;115(3):391‐399. 24078214 10.1007/s11060-013-1252-x

[41] Holtkamp N , Reuß DE , Atallah I , et al. Subclassification of nerve sheath tumors by gene expression profiling. Brain Pathol. 2004;14(3):258‐264. 15446580 10.1111/j.1750-3639.2004.tb00062.x PMC8095858

