# Supplementary figures and images for: Targeting of AKT/ERK/CTNNB1 by DAW22 as a potential therapeutic compound for malignant peripheral nerve sheath tumor
Source: Cancer Med. 2018 Aug 15;7(9):4791–800. doi: 10.1002/cam4.1732 (PMC6144169; doi:10.1002/cam4.1732)

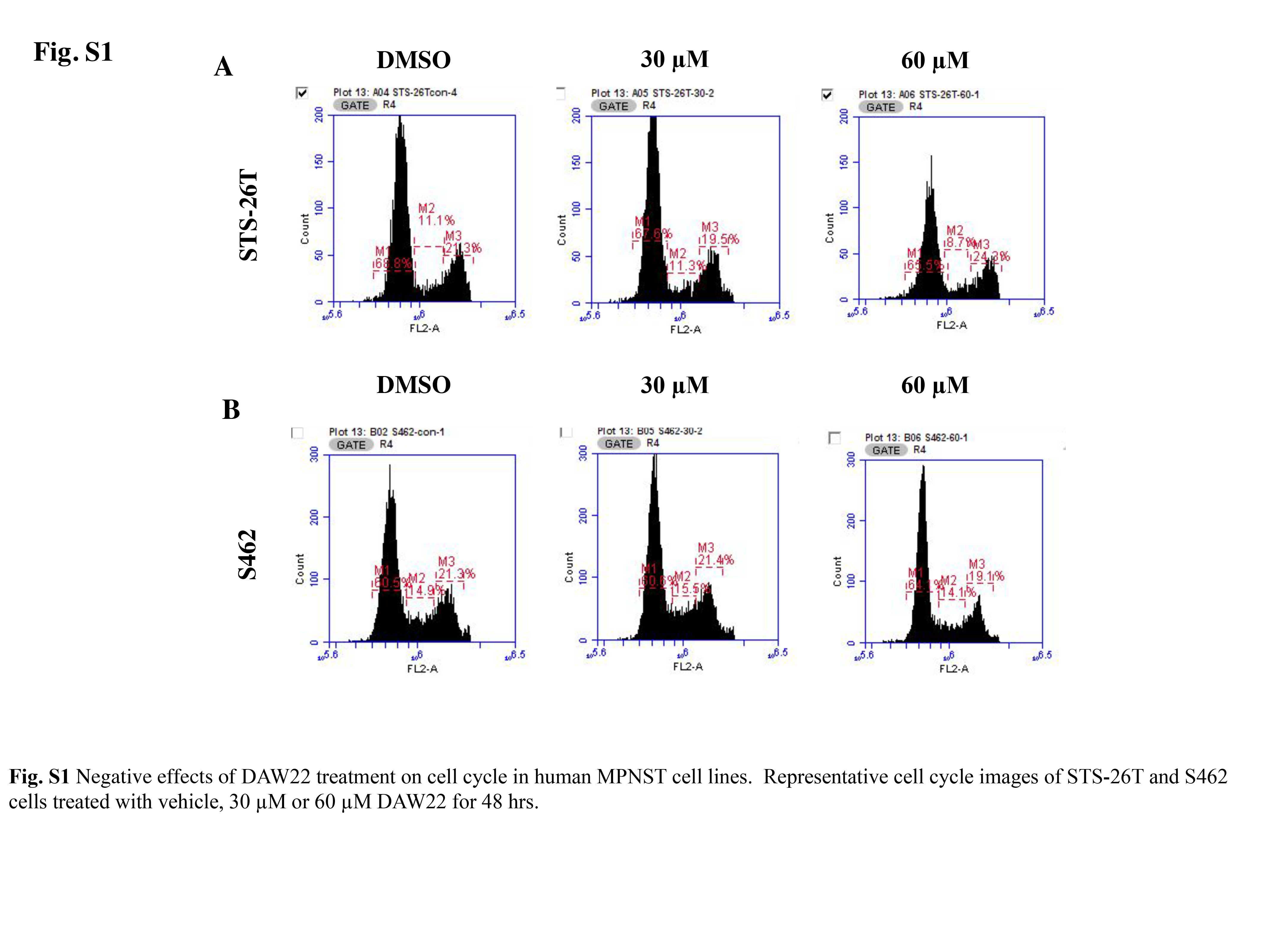

Supplement: Supplementary file 1 [file CAM4-7-4791-s001.tif]

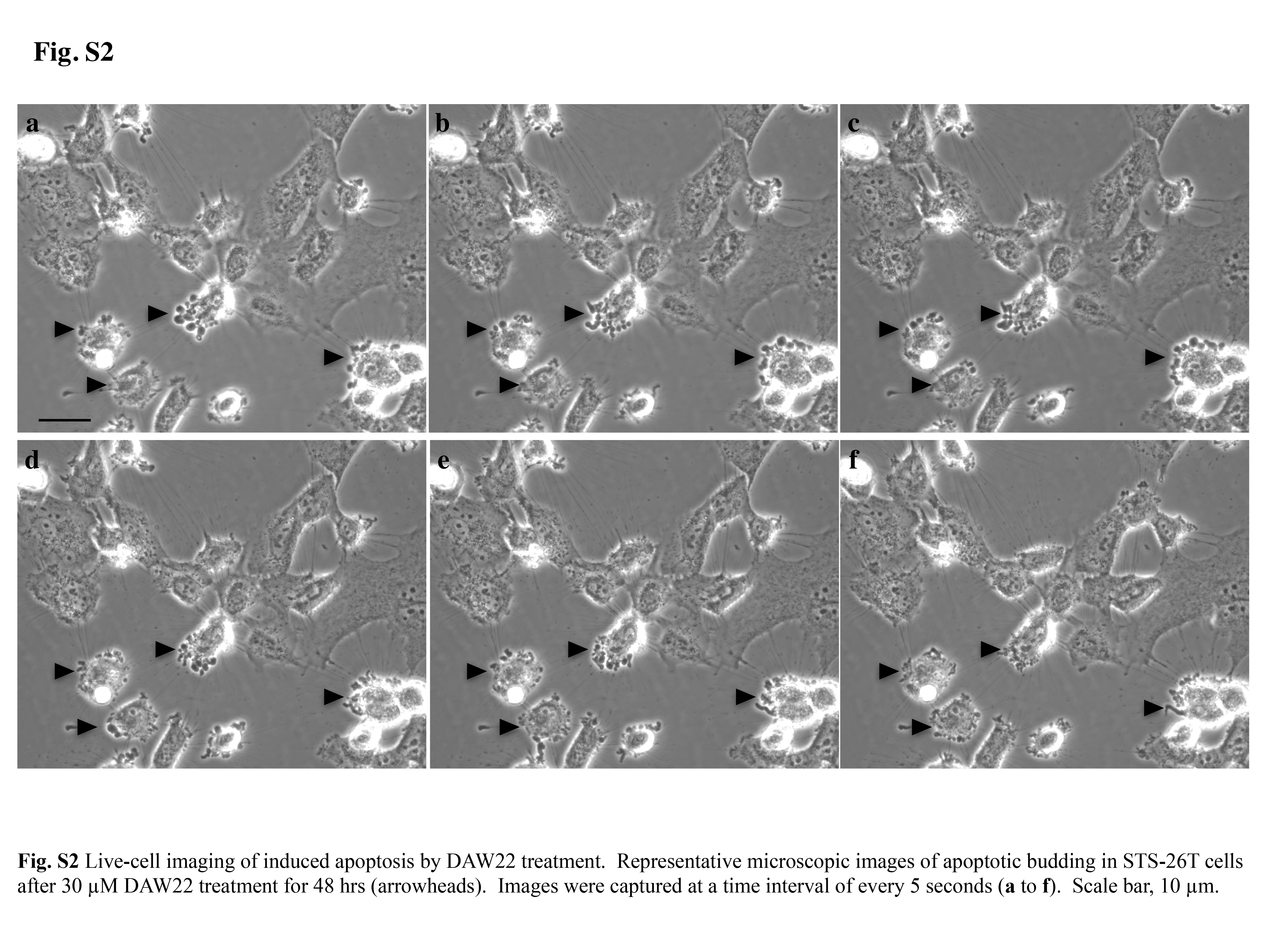

Supplement: Supplementary file 2 [file CAM4-7-4791-s002.tif]

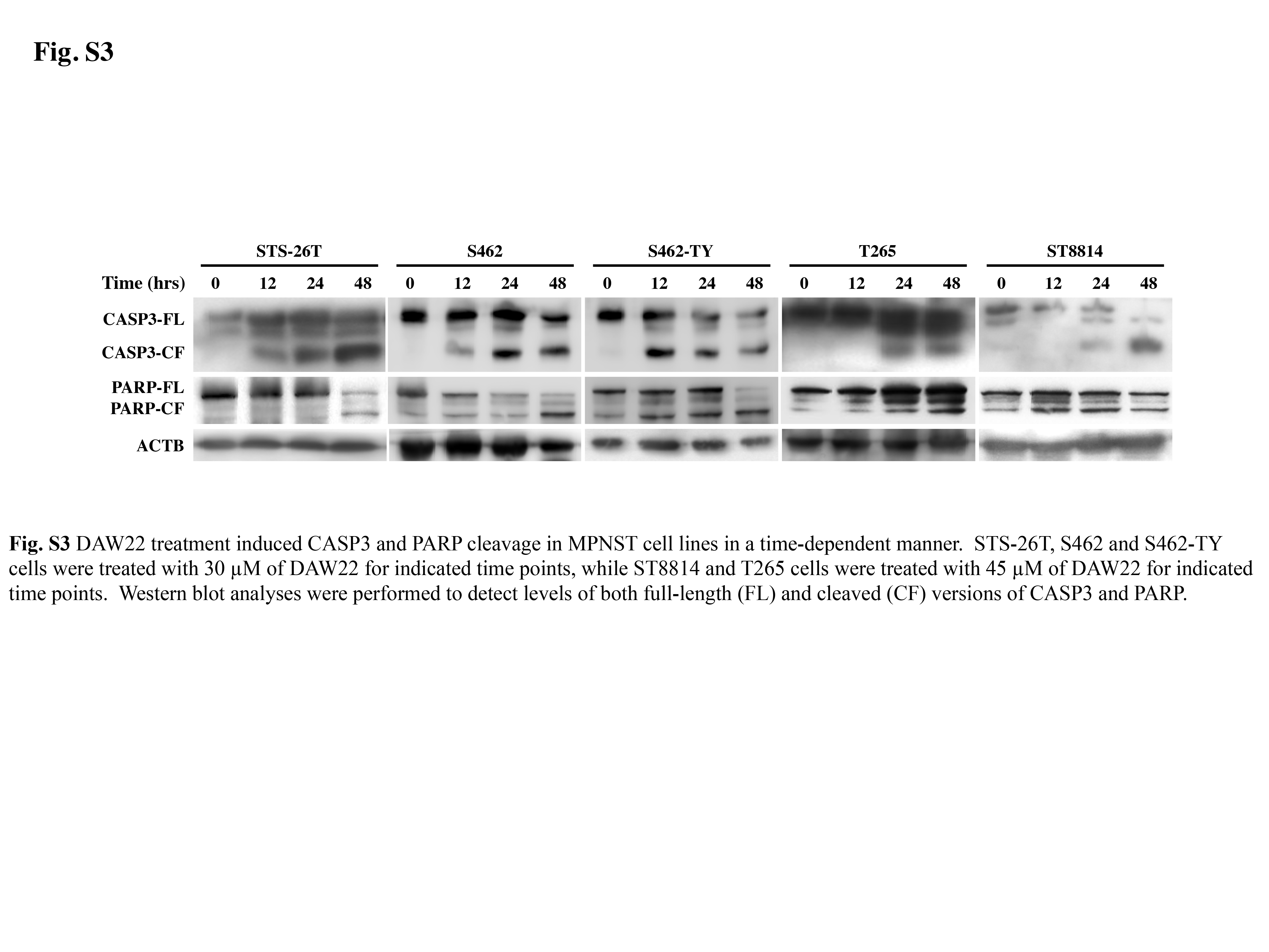

Supplement: Supplementary file 3 [file CAM4-7-4791-s003.tif]

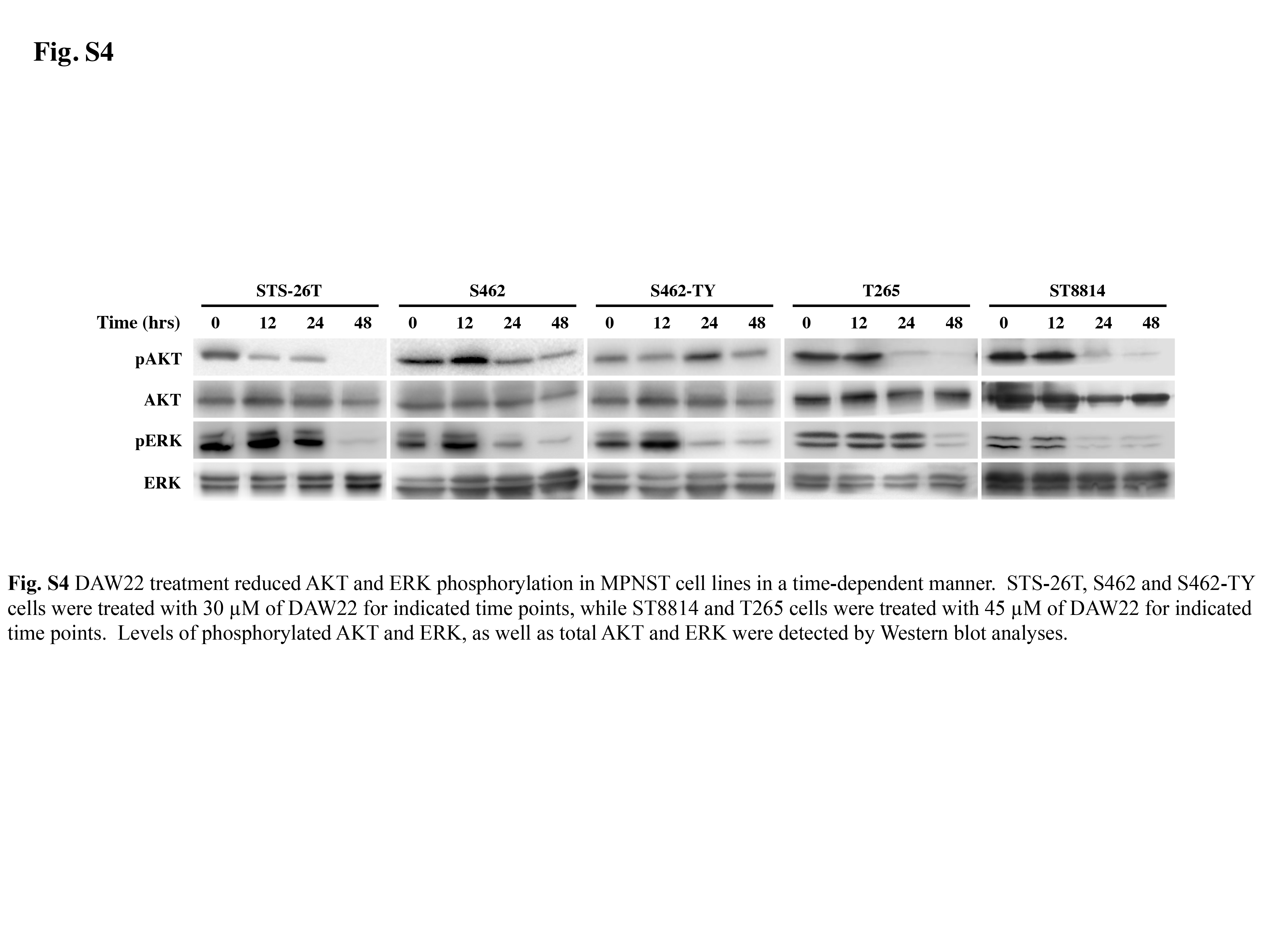

Supplement: Supplementary file 4 [file CAM4-7-4791-s004.tif]

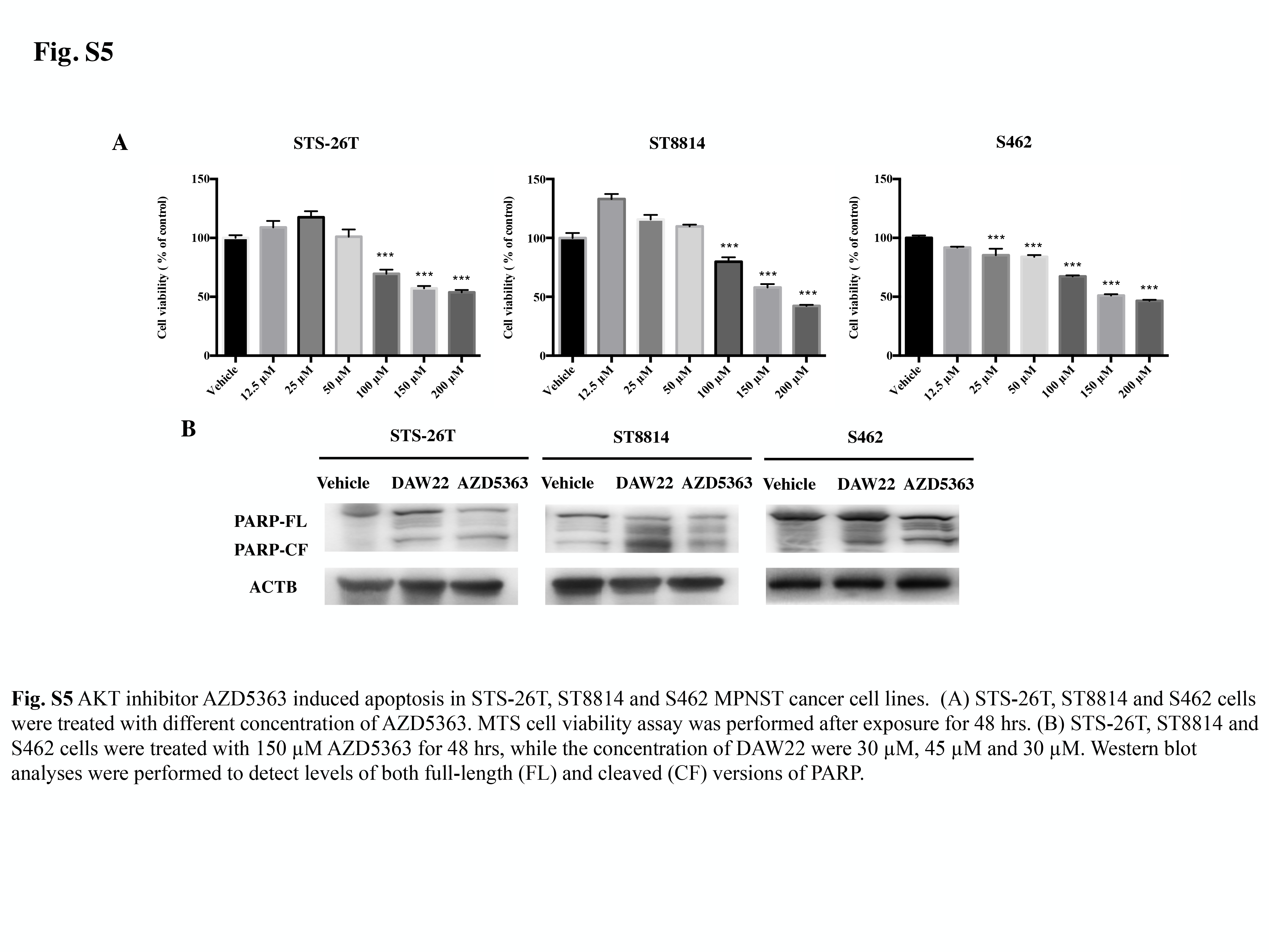

Supplement: Supplementary file 5 [file CAM4-7-4791-s005.tif]

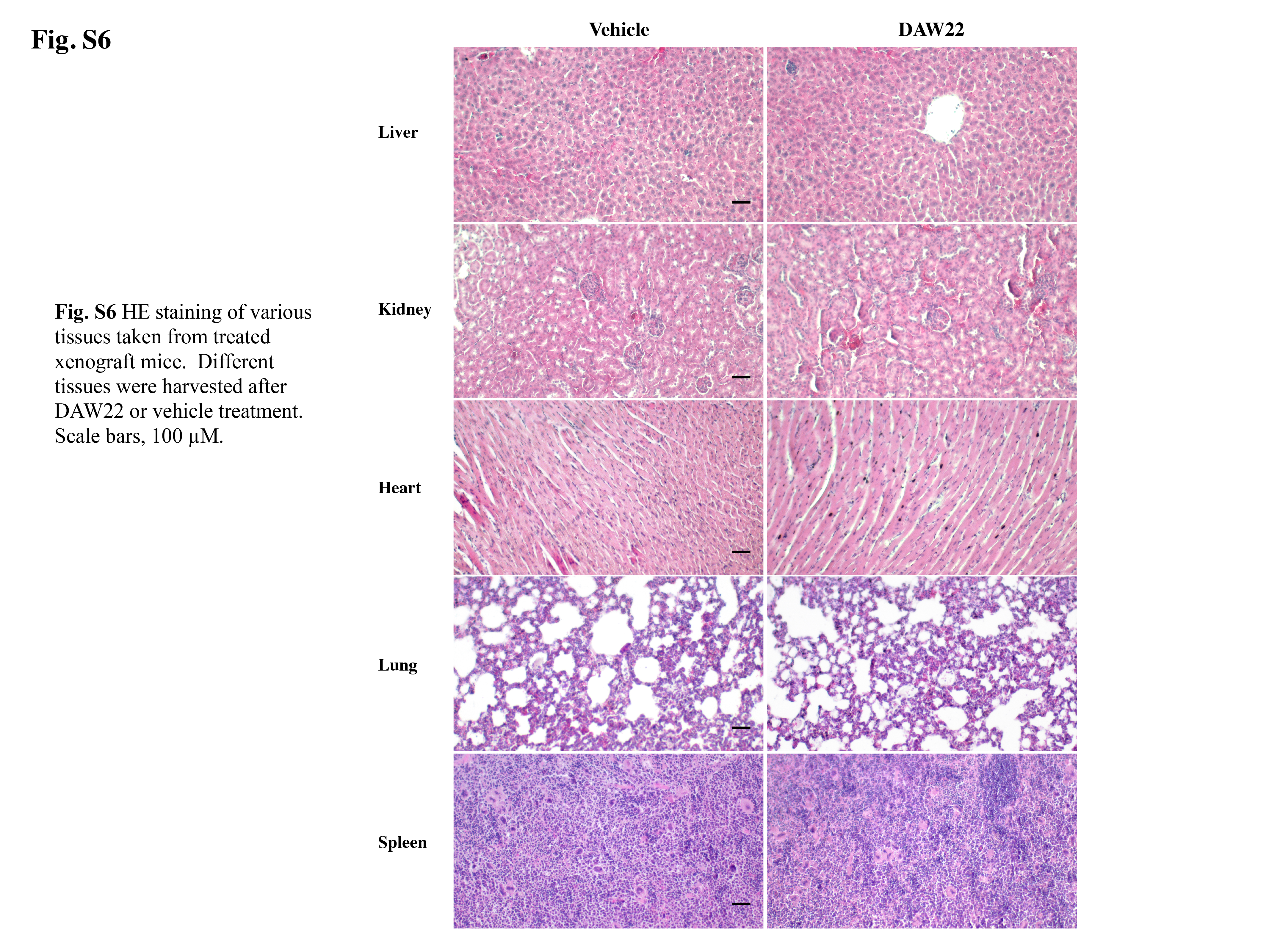

Supplement: Supplementary file 6 [file CAM4-7-4791-s006.tif]
